# Radiomics in the Setting of Neoadjuvant Radiotherapy: A New Approach for Tailored Treatment

**DOI:** 10.3390/cancers13143590

**Published:** 2021-07-17

**Authors:** Valerio Nardone, Luca Boldrini, Roberta Grassi, Davide Franceschini, Ilaria Morelli, Carlotta Becherini, Mauro Loi, Daniela Greto, Isacco Desideri

**Affiliations:** 1 Department of Precision Medicine, University of Campania “L. Vanvitelli”, 80138 Naples, Italy; v.nardone@hotmail.it (V.N.); grassi.roberta89@gmail.com (R.G.); 2 Italian Society of Medical and Interventional Radiology (SIRM), SIRM Foundation, 20122 Milan, Italy; 3 Radiation Oncology Unit, Fondazione Policlinico Universitario A. Gemelli IRCCS, 00168 Rome, Italy; luca.boldrini@policlinicogemelli.it; 4 Radiotherapy and Radiosurgery Department, IRCCS Humanitas Research Hospital, via Manzoni 56, 20089 Milan, Italy; davide.franceschini@cancercenter.humanitas.it; 5 Department of Biomedical, Experimental and Clinical Sciences “Mario Serio”, University of Florence, 50134 Florence, Italy; carlotta.becherini@libero.it; 6 Radiation Oncology Unit, Azienda Ospedaliero Universitaria Careggi, 50139 Florence, Italy; mauro.loi82@gmail.com (M.L.); gretod@aou-careggi.toscana.it (D.G.); isacco.desideri@unifi.it (I.D.); 7 Department of Experimental and Clinical Biomedical Sciences “Mario Serio”, University of Florence, 50134 Florence, Italy

**Keywords:** radiomics, neoadjuvant radiotherapy, texture analysis

## Abstract

**Simple Summary:**

This review based on a literature search aims at showing the impact of Texture Analysis in the prediction of response to neoadjuvant radiotherapy and/or chemoradiotherapy. The manuscript explores radiomics approaches in different fields of neoadjuvant radiotherapy, including esophageal cancer, lung cancer, sarcoma and rectal cancer in order to shed a light in the setting of neoadjuvant radiotherapy that can be used to tailor the best subsequent therapeutical strategy.

**Abstract:**

Introduction: Neoadjuvant radiotherapy is currently used mainly in locally advanced rectal cancer and sarcoma and in a subset of non-small cell lung cancer and esophageal cancer, whereas in other diseases it is under investigation. The evaluation of the efficacy of the induction strategy is made possible by performing imaging investigations before and after the neoadjuvant therapy and is usually challenging. In the last decade, texture analysis (TA) has been developed to help the radiologist to quantify and identify the parameters related to tumor heterogeneity, which cannot be appreciated by the naked eye. The aim of this narrative is to review the impact of TA on the prediction of response to neoadjuvant radiotherapy and or chemoradiotherapy. Materials and Methods: Key references were derived from a PubMed query. Hand searching and ClinicalTrials.gov were also used. Results: This paper contains a narrative report and a critical discussion of radiomics approaches in different fields of neoadjuvant radiotherapy, including esophageal cancer, lung cancer, sarcoma, and rectal cancer. Conclusions: Radiomics can shed a light on the setting of neoadjuvant therapies that can be used to tailor subsequent approaches or even to avoid surgery in the future. At the same, these results need to be validated in prospective and multicenter trials.

## 1. Introduction

Radiotherapy (RT) represents one of the most effective anticancer agents, which can be used either alone or in combination with other strategies (surgery, chemotherapy, and immunotherapy). It aims either to cure the patient (curative radiotherapy), to reduce the risk of locoregional relapse after surgery (adjuvant radiotherapy), to facilitate and to improve the results of surgery (neoadjuvant radiotherapy), or to relieve symptoms if a cure is not achievable (palliative radiotherapy). A neoadjuvant strategy, specifically, represents a type of induction therapy that is usually given as a first step to shrink a tumor before the main treatment, usually surgery, is given. In this setting, radiotherapy can be used either alone or in combination with chemotherapy.

This strategy is currently used mainly in locally advanced rectal cancer and sarcoma and in a subset of non-small cell lung cancer and esophageal cancer, whereas in other diseases it is still under investigation [1,2,3,4,5,6,7]. An evaluation of the efficacy of the induction strategy is made possible by performing imaging investigations before and after the neoadjuvant therapy. The imaging is evaluated by the radiologist and the response is usually classified following the RECIST guidelines [8,9].

In the last decade, texture analysis (TA) has been developed in order to help the radiologist to quantify and identify the parameters related to tumor heterogeneity that cannot be appreciated by the naked eye [10,11,12,13]. This analysis can use multiple mathematical models that are aimed to provide quantitative parameters within a selected image, which are called texture features. TA is performed employing a computer quantification of both the gray-level intensity and position of the pixels, and its use is being investigated in several fields [10,11,14,15,16,17,18,19,20,21,22,23,24,25,26,27]. More recently, a different approach to TA was developed, taking into consideration the variations in TA parameters at different acquisition times. This approach is usually called delta texture analysis or delta radiomics (D-TA) [28,29,30,31]. With this method, it is possible to investigate the role of TA variations after therapy (usually neoadjuvant chemotherapy or radiotherapy) or shortly after the beginning of the therapy.

Herein, we will discuss the impact of TA in the prediction of response to neoadjuvant radiotherapy and or chemoradiotherapy, focusing on rectal cancer, sarcoma, lung cancer, and esophageal cancer. Following a literature search, we will provide a narrative overview of these topics.

## 2. Materials and Methods

### 2.1. Evidence Acquisition

An electronic literature search was conducted in the PubMed database for English articles published up to 30 April 2021. Boolean operators (OR, AND) were used to combine the following search terms: “texture analysis”, “radiomics”, “neoadjuvant”, “neoadjuvant radiotherapy”, “induction radiotherapy”, “rectal cancer”, “sarcoma”, “esophageal cancer”, and “lung cancer”. Two independent reviewers (V.N. and L.B.) screened the titles and abstracts and performed the final article selection. Any discrepancy was resolved by discussion with a third reviewer (I.D.). Meeting proceedings (European Society of Medical Oncology—ESMO; European SocieTy for Radiotherapy and Oncology—ESTRO; American Society of Clinical Oncology—ASCO; and American Society for Radiation Oncology—ASTRO), trial registries (ClinicalTrials.gov), reference lists of published studies, review articles, and relevant books were also considered.

### 2.2. Texture Analysis: Workflow and Definition

Radiomics aims at the extraction of quantitative parameters from medical imaging. Different imaging techniques can be used for this purpose (CT, MRI, PET/CT, and US) and the features extracted can be also correlated with other cancer characteristics, such as tumor markers, metabolic activity of the tumor volume, tumor microenvironment, and tumor infiltrating lymphocytes. The integration of radiomic features and known clinical parameters with approaches such as radiogenomics is particularly important to better understand the clinical meaning of texture parameters [10,11,12,13].

Radiomics can be applied to both solid and non-solid tumors, although in the latter the gross tumor volume cannot be contoured and the texture analysis would include other endpoints (such as chemotherapy or radiotherapy toxicity or organ invasion diagnosis) [32,33]. 

The texture features can be predefined (feature-based radiomics) or identified and generated by computational models (deep learning-based radiomics) [10,11,14,15,16,17,18,19,20,21,22,23,24,25,26,27]. A feature-based radiomics workflow usually relies on image preprocessing and region-of-interest (ROI) segmentation, followed by feature extraction. Radiomics features are based on a huge number of different mathematical operations; thus, a large number (even more than 1000) of features can be extracted from a single ROI. These features can be divided in different subgroups, as follows:

(a) *Shape features*: these features refer to the geometric properties of the ROI (volume, diameter, sphericity, and compacity);
(b) *Histogram-based features:* these features are calculated from the general histogram of the Hounsfield Unit (HU) of the ROI, such as the mean, median, skewness, and kurtosis. These features do not consider the spatial orientation of the voxels;
(c) *Second-order texture features:* these features consider the statistical relationship between neighboring voxels or groups of voxels within the segmented lesion. These features can be extracted from several matrices, such as the gray-level co-occurrence matrix (GLCM), gray-level run-length matrix (GRLLM), and neighborhood gray-level different matrix (NGLDM);
(d) *Higher-order texture features:* these parameters use additional image filters, using specific mathematical transformations that can highlight specific aspects of the ROI. The filter used can be wavelet or Fourier transforms, fractal analysis, and Laplacian of Gaussian;

Deep learning–based radiomics, conversely, use neural networks or auto-encoders to generate and identify important features from input data [34]. Deep learning is an application of Artificial intelligence (AI) that represents a set of computational algorithms that are able to learn the patterns in the data provided in order to make predictions on other data sets. The application areas include image segmentation and phenotyping, radiomic signature discovery, clinical outcome prediction, and many other processes. With this AI approach, radiomics can handle a massive amount of data and at the same time can directly analyze the imaging to design the proper radiomic features.

## 3. Results

### 3.1. Texture Analysis in Neoadjuvant Radiotherapy—A Focus on Esophageal Cancer

A trimodal approach with neoadjuvant chemoradiotherapy (nCRT) followed by surgery is the treatment of choice in locally advanced esophageal cancer (EC).

A clinical evaluation of the response is made with different imaging techniques (MRI, CT, and PET) and can classify the response according to the RECIST criteria [35,36,37,38,39,40]. With these premises, radiomic analysis has been used in this setting with the aim to predict the response of the neoadjuvant strategy [37].

In patients treated for locally advanced EC, temporal changes in tumor volume (before and after nCRT) were correlated with pathological response, although the predictive value of this parameter was modest, with no correlation with overall survival [35].

Sun et al. [37] found that several histogram parameters calculated on DCE-MRI can be used in evaluating and predicting the nCRT response.

CT images are mostly used to extract morphologic information of EC, but recent studies suggest that quantitative image features can provide additional information correlated to tumor response and prognosis [41,42,43].

On the other hand, metabolic imaging has been found to be more accurate in evaluating the response to nCRT. Most 18F-FDG PET studies in EC quantify metabolic tumor activity solely by using the maximum standardized uptake value (SUVmax) [44,45]. However, this approach does not characterize the total activity nor heterogeneity of the 18F-FDG uptake for the entire tumor [46,47]. Recent studies suggest that spatial image information provides additional information than SUVmax [48,49,50,51].

It has been hypothesized that tumors could be rendered more homogeneous following treatment due to a reduction in cellular density and interstitial pressure and to normalization of the vasculature with improved intra-tumor perfusion and oxygenation [43]. Studies that performed imaging before and after treatment reported that tumor heterogeneity generally decreased following treatment [43,52]. Simoni et al. found that metabolic TV (MTV) in poor responders was significantly higher than in good responders (38.6 mL vs. 17.7 mL, *p* = 0.02) [53].

Yip et al. reported that delta-radiomics calculated within MTV60% were the least correlated with pathological response. Instead, temporal changes in tumor 18F-FDG distribution after nCRT assessed by delta-second-order radiomics (ΔRLM textures) were correlated to the OS [54].

In a study of Beukinga et al., the most predictive textural features were Long Run Low Gray-Level (LRLGLe)-PET and Run-Percentage (RP)-CT. LRLGLe-PET depends on long runs (coarse texture) with low gray levels and was higher (i.e., low and homogeneous 18F-FDG uptake) for complete responders and lower (i.e., high and heterogeneous 18F-FDG uptake) for incomplete responders, possibly due to tumor hypoxia and necrosis [55].

In a French study, Hatt et al. assessed the value of MTV, entropy, dissimilarity, high-intensity large-area emphasis, and zone percentage for the prediction of OS of 112 patients with EC who underwent definitive CRT or nCRT followed by surgery [56]. MTV and heterogeneity had a less prognostic value in EC (vs. non-small cell lung cancer), which was attributed to smaller overall volumes. The local dissimilarity parameter appeared most predictive for OS.

Current research is focused on a correlation between the biological tumor markers from pre-treatment tumor biopsies and F-FDG PET-based radiomic features. The prediction model from Beukinga et al. tried to correlate HER2 and the CD44 expression and F-FDG PET-based radiomic features in predicting a complete response (pCR) to nCRT; it showed promising ability to identify pCR [57]. Analysis of texture within tumors on medical imaging, such as CT, MRI, and PET, is emerging as a potential biomarker to predict prognosis and treatment response in patients with EC. These findings could increase our capability to predict and evaluate the nCRT response and optimize treatment planning.

### 3.2. Texture Analysis in Neoadjuvant Radiotherapy—A Focus on Lung Cancer

Lung cancer represents the leading cause of cancer death and its treatment rely on different strategies, often used in combination, such as radiotherapy, surgery, chemotherapy, and immunotherapy [58,59,60,61]. Medical imaging is pivotal in all the different phases of lung cancer management, from diagnosis to the evaluation of the efficacy of different approaches, as well as in the subsequent follow-up [62,63,64,65,66,67,68,69].

A significant body of literature is available regarding the role of textural analysis in lung cancer. Radiomic features have been used both for diagnosis (discrimination of malignant nature of lung nodules, histology prediction, and genomic analysis) and for outcome prediction, particularly after definitive chemo-radiotherapy (CT-RT) or stereotactic body radiation therapy (SBRT) [70,71,72,73,74,75,76,77,78,79]. On the other hand, few studies focused on neoadjuvant radiotherapy, possibly because of the controversial benefit of adding surgery after CT-RT [80,81]. The largest available series included 127 stage II–III NSCLC patients treated with neoadjuvant chemoradiotherapy (CT-RT) followed by surgical resection. Texture analysis was conducted on primary tumors with features extracted from planning CT. The endpoint of the analysis was a pathologic response and gross residual disease. Seven features (one shape feature, three second-order features, and three higher-order features) were predictive for pathologic gross residual disease (AUC > 0.6, *p*-value < 0.05) and one for pathologic complete response (one higher-order feature) (AUC = 0.63, *p*-value = 0.01). These features suggested that a spherical disproportionality of the primary tumor site (i.e., more complex shape) was a predictor of pathological response, while spherical tumors and tumors with large flat zones were more likely to be linked with residual disease [82]. The same group conducted another analysis on a similar population, analyzing also features from pathologic lymph nodes. Radiomics analysis was performed on 85 primary tumors and 178 lymph nodes with the same endpoints as above (pathologic response and gross residual disease). A mix of clinical variables and conventional and radiomic features were used to create a predictive model. The model built from the radiomic features was the best at predicting pathologic response (AUC 0.68, *p* < 0.05), while a mixed clinical and radiomic model was better performing for gross residual disease (AUC 0.73, *p* < 0.05). The authors concluded that the features extracted from the lymph nodes were more informative than those derived by primary tumors [83].

A small study analyzing PET-CT included 13 patients on a prospective clinical trial of trimodal therapy for resectable locally advanced NSCLC. A true texture analysis was not performed; however, pre- and post-chemo-radiotherapy SUV was not correlated with any clinical endpoint (pathologic complete response, PFS, and OS) [84].

Chong et al. performed a texture analysis on a mixed population with NSCLC treated with neoadjuvant chemo-radiotherapy (CT-RT) or neoadjuvant EGFR-inhibitor. Focusing on the CT-RT group, 28 patients were included in the study. A pre-operative CT was used for analysis, and the endpoint again was a pathologic response. In the univariate analysis, tumor volume, mass, kurtosis, and skewness were significant predictors of pathologic response, although only kurtosis maintained its significance in the multivariate analysis (OR 1.107, *p* = 0.009). ROC analysis showed that the AUC for kurtosis was 0.943 and that the optimal cut-off value of percent change of kurtosis for predicting pathologic response was less than −23 (sensitivity, 87.5%; specificity, 84.3%) [85].

More recently, Khorrami et al. conducted a textural analysis on 90 stage IIIA NSCLC patients treated with neoadjuvant CT-RT, followed by surgical resection. Pretreatment CT scans were used for features extraction. Patients were randomly split into two sets, one for training and one for testing.

Interestingly, the authors analyzed the features not only from primary tumors, but also from the peritumoral region (defined as a 15 mm dilation from primary nodule) to identify a possible predicting role of the tumor microenvironment.

Thirteen intratumoral and peritumoral radiomic texture features were found to be predictive of major pathologic response and were used to define a classifier with an AUC of 0.90 ± 0.025 within the training set, and an AUC = 0.86 in the test set. This signature was also predictive for OS (HR = 11.18, 95% CI = 3.17, 44.1; *p*-value = 0.008) and DFS (HR = 2.78, 95% CI = 1.11, 4.12; *p*-value = 0.0042) in the testing set. The combination of peritumoral and intratumoral features performed better than a clustering built only on intratumoral features.

The results of this study highlight that heterogeneous enhancement and disruption of textural patterns within and outside the nodules (better identified by Law Laplacian and Laws features) can predict not only response, but also patients’ prognosis [86].

Identifying patients who respond completely to chemoradiation and who do not require additional invasive local therapy, also considering the controversial role of surgery in this scenario, is an unmet clinical need.

The intrinsic potential of texture analysis in predicting a response after neoadjuvant CT-RT is plain. However, available studies are not enough to consider radiomics as a ready and easily accessible clinical biomarker. Larger studies, with a higher number of patients and with external validation, are needed before radiomic analysis could be clinically implemented. Analyzing available data, a special attention in future research should be paid to nodal and peritumoral features, which are potentially even more predictive than primary tumor analysis.

### 3.3. Texture Analysis in Neoadjuvant Radiotherapy—A Focus on Sarcoma

Soft tissue sarcomas (STS) are a group of mesenchymal malignancies encompassing a wide array of distinct clinical entities, including over 50 different histological subtypes [87]. Due to their rarity and non-specific clinical presentation (mostly consisting of slowly-growing indolent swellings in limbs or trunk), a differential diagnosis with benign tumors is challenging and may result in a significant delay to curative treatment, consisting of wide surgical excision in absence of metastatic dissemination [87,88]. Furthermore, their intrinsic heterogeneity is also displayed by variable levels of genomic profile complexity and structural architecture, resulting in different clinical presentations and inconsistent responses to standard treatments [88,89,90]. Unfortunately, one among the major predictors of outcome in operable patients—tumor histological grading—may be underestimated by tumor biopsy [91]. This is a relevant issue, since tumor grade determination may guide treatment management: low-grade STSs may be treated with planned marginal excision, sparing patients from more extensive surgery [92], while detection of high-grade disease may serve as a decision criteria for pre-operative treatment intensification [93,94]. Moreover, while the additional benefit of adjuvant chemotherapy is questioned and limited to a subset of high-risk patients, no consensual criteria have been provided to define this population; clinical [95] and genomic [96] classifiers have been proposed, although their use have not been implemented in clinical practice yet.

Hence, there is an urgent need to identify novel and readily available biomarkers to improve diagnosis, predict the disease course, and possibly define a tailored pattern of care in patients at high risk of disease relapse [88]. Radiomics may provide a valuable source of information in STS patients. Due to its non-invasive nature, radiomics may quickly discern benign from malignant lesions, timely addressing the need for further investigation; secondarily, it may provide an appropriate histopathologic grade determination on the whole tumor bulk, thus overcoming the limitations of tumor biopsy in accounting for intratumor heterogeneity; finally, identification of specific signatures correlated with outcome may allow prediction of relapse risk and eligibility for further interventions.

Improvement in differential diagnosis of malignancy have been reported by several authors [97,98]. In particular radiomics-based differentiation between soft-tissue lipoma and well-differentiated liposarcoma [99,100] was demonstrated despite similar radiologic and pathologic presentation, often requiring molecular analysis of MDM2 amplification status; interestingly, superior performance of a machine-learning classifier as compared to trained radiologists has been shown [101]. Similarly, radiomic features allowed to distinguish myxoma from myxofibrosarcoma [102] and atypical leiomyoma from uterine sarcoma [103,104,105].

Machine-learning algorithms proved also useful in the prediction of histopathologic grade. Using CT- or MRI-based radiomics, different signatures associated with high-grade disease were identified [106,107,108,109,110,111,112]; in some cases, integration with clinical features resulted in the establishment of a prognostic nomogram for risk stratification [108].

A radiomic approach may be used to improve prediction of patients’ outcome. RM texture analysis alone [113,114] or combined with PET/CT metabolic data [115,116] was associated with metastatic relapse and specific signatures were identified for prediction of survival [117,118]. Radiomic analysis was also applied on surveillance MRI in patients undergoing follow-up after surgical resection [119], resulting in improved detection and characterization of local recurrence [120].

Concerning prediction of sensitivity to treatment, preliminary results reported adequate concordance between radiomic features and response to neoadjuvant chemotherapy [121] or chemoradiation [122], as well as exclusive chemotherapy in unresectable patients [119,123].

On the other hand, while radiomics analysis correlated with adipocytic maturation following neoadjuvant chemotherapy in myxoid/round cells liposarcomas, it occurred independently from the chemotherapy regimen and was not correlated with metastasis-free survival [124]; similarly, radiomics was insufficient to predict the response from hypofractionated pre-operative RT [125].

Despite promising preliminary results, no radiomic signature for sarcoma has been implemented to date in clinical practice. Multiple issues have been detected in the pathway to translation from exploratory analysis into standard of care, ranging from lack of external validation and replicability, consistency, and cost-effectiveness of the imaging biomarkers proposed at present [126]. While these shortcomings are partially motivated by the complex workflow and relevant technical requirements needed for the development and elaboration of radiomic signatures, further effort is required for successful translation into clinical use.

### 3.4. Texture Analysis in Neoadjuvant Radiotherapy—A Focus on Rectal Cancer

Adequately predicting the response to neoadjuvant chemoradiotherapy (nCRT) in rectal cancer represents a key factor in order to achieve a fully personalized treatment approach, especially in the case of locally advanced disease presentation, which constitute a well-established indication for radiation therapy [127]. The identification of biomarkers able to predict patients’ outcomes (i.e., response to nCRT) currently represents a priority in the radiotherapy research landscape, and imaging-derived response predictors are a topic of active investigation [128,129,130,131,132,133].

Thanks to the extensive use of MRI as a staging imaging technique for this specific disease, the imaging community rapidly focused its research efforts on it and the first preliminary observations have been published since the early 2000′s. These first studies generally aimed to correlate the response to neoadjuvant treatments with the Gross Tumor Volume’s (GTV) morphological modifications, mainly taking advantage of the high image quality provided by MRI [134,135,136] and also proposing to address the metabolic information disclosed by PET-CT staging imaging [137,138].

The recent development of TA and radiomics-based image analysis have introduced new predictive modelling techniques and offered promising insights for quantitative imaging characterization of rectal cancer, when compared to the mere volumetric assessment [139].

Most of the papers available in the literature focus on the response to nCRT prediction, using several imaging and modelling techniques. Handcrafted radiomics is used in the largest majority of these studies, achieving overall satisfactory results even when compared to more advanced machine learning approaches (i.e., deep learning) [34].

Nevertheless, the obtained level of evidence is still unfortunately low and inconclusive, mainly due to the overall low quality of these studies, as pointed out by two recent reviews that highlight the limited replicability and reproducibility of the available models [140,141].

Furthermore, the largest majority of the published papers does not meet the minimal technical quality criteria, such as the ones proposed by the RQS and QUADAS-2 standards [142,143]. In this context, it has also to be said that this issue suffers from the initial enthusiasm that researchers have addressed to radiomics and TA analysis, publishing several exploratory papers on very limited cohorts of patients and often using non-standardized, and thus not reproducible analysis pipelines.

Furthermore, only few models are based on a properly selected reduced number of features that make their applicability easier and more straightforward, while the majority gathers hundreds of variables.

Among the different imaging techniques used, it is evident that staging MRI represents the one of choice for TA and radiomics studies in general, primarily thanks to the large availability of images and to the prominent current role fulfilled by MRI in the framework of the international rectal cancer management guidelines as the imaging gold standard for disease staging [144].

Response to therapy (e.g., tumor downstaging and pathological complete response, pCR) represents the most common outcomes of these models, whose main part focuses on the use of T2w and Diffusion Weighted Imaging (DWI), while the use of Dynamic Contrast Enhanced (DCE) images is still less common [141].

Interestingly, the largest majority of the published MRI radiomics studies takes into account histogram features (considered alone or in more advanced models based also on textural, shape, and filtered ones), supporting systematic investigations in this direction [28,145,146,147,148,149,150,151,152,153,154,155,156,157,158,159,160,161,162,163,164,165,166,167,168,169,170,171,172].

Unfortunately, no consensus has been reached in the literature about the role and the potential biological correlates of the radiomics features that have been identified in the studies published so far. In their recent review, Staal and colleagues systematically point out this pitfall, shedding a light on the complex translational impact of the published observations, but also pointing out interesting correlations that deserve to be further explored, such as the one between tumor heterogeneity, image homogeneity, and generally favorable patients’ outcomes [141].

Besides traditional diagnostic imaging, the recent introduction of MR-guided radiotherapy (MRgRT) paves the way to innovative clinical approaches, including promising modelling applications for hybrid imaging TA and radiomics analysis [173,174,175]. Although the published evidence is still scarce and exploratory, rectal cancer hybrid imaging appears indeed to have significant potentialities for the set-up of radiomics-based prediction models. A first hypothesis generating experience identified two delta radiomics features (ΔL_least and Δglnu) as possible candidate predictors of a complete response (cCR) after rectal cancer neoadjuvant therapy using 0.35 T setup images [28]. The model was then externally validated using independent cohorts and achieved remarkable performances for both cCR and pCR (accuracy for cCR prediction: ΔLleast = 81% and Δglnu = 63%; accuracy for pCR prediction: ΔLleast 79% and Δglnu = 40%), confirming the interest in applying radiomics to hybrid MR images [150]. These observations may lead to a new generation of innovative trials, personalizing a patient’s treatment based on imaging-based prediction results (clinical trials NCT04815694).

Besides MRI-based investigations, a more limited number of papers explore the performances of PET-CT for response prediction, obtaining overall unsatisfactory results in terms of prediction performances, probably due to technical reasons relative to PET imaging formats that still remain unsolved [176,177,178,179,180].

Radiomics and TA therefore represent a promising field of investigation for rectal cancer and may support the continuous development of accurate prediction models, integrating the variables extracted from different omics domains in a holistic and fully personalized treatment approach [181,182].

## 4. Future Directions and Conclusions

All the above-mentioned studies have shown the promising direction of using radiomics in the field of neoadjuvant therapies. Despite an impressive number of retrospective studies, the number of prospective clinical trials investigating the potential role of texture analysis in neoadjuvant radiotherapy remains inexplicably low (see Table 1).

There still are major limitations to solve before TA can be successfully applied in the clinical management of cancer patients. More specifically, the current major pitfalls in TA are the lack of image standardization, the lack of feature extraction standardization, the problems related to data sharing, and the distrust of the clinicians in the black box approach.

All the variations in scanning devices, acquisition protocols, and parameters of reconstructions may impact on the process of feature extraction and analysis. Although different standardization techniques may calibrate and overcome partially this problem [183,184,185,186], the ideal solution is to standardize the image acquisition in dedicated prospective trials.

Similarly, the process of feature extraction is often challenging and can lead to several biases that impact the reliability of the TA parameters among different institutions. Hopefully, with the help of artificial intelligence and the optimization of machine learning approaches, the limitations of feature extraction (ROI segmentation, feature selection, and endpoint correlation) should be overcome in the foreseeable future [187,188,189,190,191].

At the same time, sample size in TA research represents a drawback in current research, as most studies cited above cover a limited number of patients. The use of big data, instead, could overcome this problem, and could ensure a higher statistical significance in the interpretation of the data. Thus, several efforts should be made in the development of high-number and high-quality shared databases in the future. These datasets require joint efforts by both companies and institutions, such as the Cancer Learning Intelligence Network for Quality and Flatiron Health and the Cancer Imaging Archive. At the same time, the development and maintenance of such shared databases require high standards of security, in order to respect patients’ privacy and actual law order in terms of data sharing and data privacy.

The texture parameters are somehow difficult to describe and to refer to known clinical variables; as a consequence, TA looks like a black box to clinicians, as the connection between the TA parameters and the endpoints is not fully understood. Nonetheless, more should be done in order to correlate radiomics to the underlying clinical and molecular connotation with approaches such as radiogenomics; at the same time, more should be learned about artificial intelligence and radiomics by the clinicians [192,193].

Currently, many researchers are investigating methods to diminish the black box perception [194]. These two processes together can help to empty the gap and to remove the black box uncertainties, in order to promote TA towards clinical practice.

Despite these pitfalls, TA analysis can still aspire to become an optimal surrogate biomarker. In this context, research efforts should be concentrated precisely on the field of neoadjuvant therapies. In this setting, in fact, it is easier to test different approaches because the endpoint is usually the pathological response, which is more immediate to accomplish. In this field, the clinicians could trust more easily in radiomics, if they are called to test an effective and reliable model. For this reason, neoadjuvant therapy represents the ideal playground for TA and a higher number of prospective trials should be ideated and conducted in the next future.

Finally, future combination of radiomics analysis in the setting of immunotherapy is needed in the next years. This interesting challenge is particularly important, as imaging is always used in the clinical management of cancer patients, and TA may in the future provide useful information regarding the molecular characteristics of cancer, which may differ from a biopsy due to the accumulation of multiple genetic mutations and epigenetic alterations.

## Figures and Tables

**Table 1:** Prospective clinical trials investigating the potential role of texture analysis in neoadjuvant radiotherapy.

NCT Number	Study Type	Cancer Type	Trial Design	Contacts and Locations	Trial Design
NCT02439086	Interventional	Rectal Cancer	18F-FDG-PET-CT and texture analysis of MRI performed 9 weeks after Neoadjuvant Chemo-radiotherapy in patients with locally advanced rectal cancer to test the ability to identify patients with Complete Response.	Medhat S Alaker, Colchester General Hospital, UK	Single arm, patients will have 2 PET CT scans: one before radiotherapy, and one 9 weeks after.
NCT04273477	Observational	Rectal cancer	Radiomics prediction model to predict the tumor response to neoadjuvant chemoradiotherapy (nCRT) before the nCRT is administered.	Xiangbo Wan, Sun Yat-sen University, China	Multicenter, prospective, observational clinical study, evaluating MRI performed before nCRT.
NCT03238885	Observational	Rectal cancer	Develop and validate a radiomics model for individualized pCR evaluation after CRT in patients. The ultimate aim is to select appropriate LARC patients for omission of surgery.	Sun Ying-Shi, Beijing Cancer Hospital, China	Prospective, observational cohort study, investigating 3T MRI performed before and after nCRT.
NCT04489368	Observational	Esophagus Cancer	Develop models to predict pCR based on pre-neoadjuvant imaging modalities	Kundan S Chufal, Rajiv Gandhi Cancer Institute & Research Center, India	Prospective, observational cohort study, investigating imaging performed before nCRT.
NCT04278274	Observational	Rectal Cancer	Evaluation of Post-Neoadjuvant Treatment MRI Based AI System to Predict Pathologic Complete Response for Patients With Rectal Cancer.	Xiangbo Wan, Sun Yat-sen University, China	Multicenter, prospective, observational clinical study, evaluating MRI performed after nCRT and before surgery.
NCT04815694	Interventional	Rectal Cancer	Investigate the impact of dose escalation in rectal cancer, identifying the poor responder cases using the early tumor regression index during the course of radiotherapy and increasing the prescribed dose in these patients. Secondary endpoint is prospective validation of delta radiomics MR-guide Radiotherapy model	Giuditta Chiloiro, Fondazione Policlinico Universitario A.Gemelli IRCCS, Italy	Interventional, two arms. In experimental arm RT Dose escalation will be performed in patients based on Early Regression Index values calculated at second week on nCRT
NCT04359732	Interventional	Esophagus Cancer	Prediction of Assessment of Response to Neoadjuvant Chemo-Radio-Therapy (nCRT) for Esophageal and Gastroesophageal Junction Cancer (GEJ) Using a Fully Integrated PET/MRI	Francesco De Cobelli, IRCCS San Raffaele, Italy	Interventional, single arm. An additional intermediate 18-FDG PET/MRI will be performed during nCRT.
NCT03237130	Observational	Esophagus Cancer	Establishment of an image feature extraction and selection method for identifying lymph node metastasis of esophageal cancer	Ying-Shi Sun, Dept.Radiology, Peking University Cancer Hospital, China	Prospective, observational cohort study. Each patient will receive preoperative enhanced chest CT examination, and their CT images will be used for analysis
NCT04090450	Observational	Rectal Cancer	Retrospective study using images acquired routinely for diagnosis of rectal cancer to see if these could be used to predict responses to radiotherapy treatment and if it can, whether the treatment can be optimized to produce better outcome for patients.	Peter Mbanu, University of Manchester, UK	Retrospective, observational cohort study and will recruit patients who have had nCRT for rectal cancer. MR radiomics features will be analyzed.
NCT03029793	Observational	Esophagus Cancer	Determine whether combination of molecular and biomarkers with functional imaging can predict pathologic response and clinical outcomes in squamous esophageal cancer patients who undergo trimodal therapy which includes neoadjuvant chemoradiotherapy and surgery	Li-Na Zhao, Air Force Military Medical University, China	Prospective, observational cohort study, investigating imaging performed before nCRT. The study will collect both tissue samples and imaging in locally advanced esophageal cancer patients.
NCT04207918	Interventional	Esophagus Cancer	Evaluate the 1-year local tumor control rates after the targeted therapy of intensity-modulated radiation therapy synchronized chemotherapy with nimotuzumab. Secondary texture analysis of CT and MRI simulation imaging in predicting tumor response rate is included.	Wang Xin, Chinese Academy of Medical Sciences, China	Experimental, single arm, nCRT arm receives RT concurrently with S-1 and Nimotuzumab. Texture analysis of CT and/or MRI simulation will be analyzed in predicting tumor response rate and prognosis.

## References

[1] Conroy T., Bosset J.F., Etienne P.L., Rio E., François É., Mesgouez-Nebout N., Vendrely V., Artignan X., Bouché O., Gargot D. (2021). Neoadjuvant chemotherapy with FOLFIRINOX and preoperative chemoradiotherapy for patients with locally advanced rectal cancer (UNICANCER-PRODIGE 23): A multicentre, randomised, open-label, phase 3 trial. Lancet Oncol..

[2] Bahadoer R.R., Dijkstra E.A., van Etten B., Marijnen C.A.M., Putter H., Kranenbarg E.M., Roodvoets A.G.H., Nagtegaal I.D., Beets-Tan R.G.H., Blomqvist L.K. (2021). Short-course radiotherapy followed by chemotherapy before total mesorectal excision (TME) versus preoperative chemoradiotherapy, TME, and optional adjuvant chemotherapy in locally advanced rectal cancer (RAPIDO): A randomised, open-label, phase 3 trial. Lancet Oncol..

[3] Bonvalot S., Gronchi A., Le Péchoux C., Swallow C.J., Strauss D., Meeus P., van Coevorden F., Stoldt S., Stoeckle E., Rutkowski P. (2020). Preoperative radiotherapy plus surgery versus surgery alone for patients with primary retroperitoneal sarcoma (EORTC-62092: STRASS): A multicentre, open-label, randomised, phase 3 trial. Lancet Oncol..

[4] Lorenzen S., Biederstädt A. (2020). RACE-trial: Neoadjuvant radiochemotherapy versus chemotherapy for patients with locally advanced, potentially resectable adenocarcinoma of the gastroesophageal junction—A randomized phase III joint study of the AIO, ARO and DGAV. BMC Cancer.

[5] Sun H.B., Xing W.Q., Liu X.B., Zheng Y., Yang S.J., Wang Z.F., Liu S.L., Ba Y.F., Zhang R.X., Liu B.X. (2020). Neoadjuvant chemotherapy versus neoadjuvant chemoradiotherapy for locally advanced oesophageal squamous cell carcinoma: A single-Centre, open-label, randomized, controlled, clinical trial (HCHTOG1903). BMC Cancer.

[6] Yang K.L., Chang Y.C., Ko H.L., Chi M.S., Wang H.E., Hsu P.S., Lin C.C., Yeh D.Y., Kao S.J., Jiang J.S. (2016). Optimizing Survival of Patients with Marginally Operable Stage IIIA Non-Small-Cell Lung Cancer Receiving Chemoradiotherapy with or Without Surgery. Clin. Lung Cancer.

[7] Chidley P., Foroudi F. (2021). Neoadjuvant radiotherapy for locally advanced and high-risk breast cancer. J. Med. Imaging Radiat. Oncol.

[8] Schwartz L.H., Litière S., de Vries E., Ford R., Gwyther S., Mandrekar S., Shankar L., Bogaerts J., Chen A., Dancey J. (2016). RECIST 1.1-Update and clarification: From the RECIST committee. Eur. J. Cancer.

[9] Seymour L., Bogaerts J., Perrone A., Ford R., Schwartz L.H., Mandrekar S., Lin N.U., Litiere S., Dancey J., Chen A. (2017). iRECIST: Guidelines for response criteria for use in trials testing immunotherapeutics. Lancet Oncol.

[10] Neri E., Coppola F., Miele V., Bibbolino C., Grassi R. (2020). Artificial intelligence: Who is responsible for the diagnosis?. Radiol. Med..

[11] Rossi F., Bignotti B., Bianchi L., Picasso R., Martinoli C., Tagliafico A.S. (2020). Radiomics of peripheral nerves MRI in mild carpal and cubital tunnel syndrome. Radiol. Med..

[12] Zhang L., Kang L., Li G., Zhang X., Ren J., Shi Z., Li J., Yu S. (2020). Computed tomography-based radiomics model for discriminating the risk stratification of gastrointestinal stromal tumors. Radiol. Med..

[13] Zhang Y., Zhu Y., Zhang K., Liu Y., Cui J., Tao J., Wang Y., Wang S. (2020). Invasive ductal breast cancer: Preoperative predict Ki-67 index based on radiomics of ADC maps. Radiol. Med..

[14] Coppola F., Faggioni L., Regge D., Giovagnoni A., Golfieri R., Bibbolino C., Miele V., Neri E., Grassi R. (2021). Artificial intelligence: Radiologists’expectations and opinions gleaned from a nationwide online survey. Radiol. Med..

[15] Grassi R., Miele V. (2019). Artificial intelligence: A challenge for third millennium radiologist. Radiol. Med..

[16] Nardone V., Tini P., Carbone S.F., Grassi A., Biondi M., Sebaste L., Carfagno T., Vanzi E., De Otto G., Battaglia G. (2017). Bone texture analysis using CT-simulation scans to individuate risk parameters for radiation-induced insufficiency fractures. Osteoporos. Int..

[17] Nardone V., Tini P., Croci S., Carbone S.F., Sebaste L., Carfagno T., Battaglia G., Pastina P., Rubino G., Mazzei M.A. (2018). 3D bone texture analysis as a potential predictor of radiation-induced insufficiency fractures. Quant. Imaging Med. Surg..

[18] Belfiore M.P., Urraro F., Grassi R., Giacobbe G., Patelli G., Cappabianca S., Reginelli A. (2020). Artificial intelligence to codify lung CT in Covid-19 patients. Radiol. Med..

[19] Van Assen M., Muscogiuri G., Caruso D., Lee S.J., Laghi A., De Cecco C.N. (2020). Artificial intelligence in cardiac radiology. Radiol. Med..

[20] Abdollahi H., Mofid B., Shiri I., Razzaghdoust A., Saadipoor A., Mahdavi A., Galandooz H.M., Mahdavi S.R. (2019). Machine learning-based radiomic models to predict intensity-modulated radiation therapy response, Gleason score and stage in prostate cancer. Radiol. Med..

[21] Ciolina M., Vinci V., Villani L., Gigli S., Saldari M., Panici P.B., Perniola G., Laghi A., Catalano C., Manganaro L. (2019). Texture analysis versus conventional MRI prognostic factors in predicting tumor response to neoadjuvant chemotherapy in patients with locally advanced cancer of the uterine cervix. Radiol. Med..

[22] Filograna L., Lenkowicz J., Cellini F., Dinapoli N., Manfrida S., Magarelli N., Leone A., Colosimo C., Valentini V. (2019). Identification of the most significant magnetic resonance imaging (MRI) radiomic features in oncological patients with vertebral bone marrow metastatic disease: A feasibility study. Radiol. Med..

[23] Ravanelli M., Agazzi G.M., Tononcelli E., Roca E., Cabassa P., Baiocchi G., Berruti A., Maroldi R., Farina D. (2019). Texture features of colorectal liver metastases on pretreatment contrast-enhanced CT may predict response and prognosis in patients treated with bevacizumab-containing chemotherapy: A pilot study including comparison with standard chemotherapy. Radiol. Med..

[24] Hu H., Shan Q., Chen S., Li B., Feng S., Xu E., Li X., Long J., Xie X., Lu M. (2020). CT-based radiomics for preoperative prediction of early recurrent hepatocellular carcinoma: Technical reproducibility of acquisition and scanners. Radiol. Med..

[25] Vernaleone M., Bonomo P. (2019). Robotic stereotactic radiotherapy for liver oligometastases from colorectal cancer: A single-center experience. Radiol. Med..

[26] Kirienko M., Ninatti G. (2020). Computed tomography (CT)-derived radiomic features differentiate prevascular mediastinum masses as thymic neoplasms versus lymphomas. Radiol. Med..

[27] Nazari M., Shiri I., Hajianfar G., Oveisi N., Abdollahi H., Deevband M.R., Oveisi M., Zaidi H. (2020). Noninvasive Fuhrman grading of clear cell renal cell carcinoma using computed tomography radiomic features and machine learning. Radiol. Med..

[28] Boldrini L., Cusumano D., Chiloiro G., Casa C., Masciocchi C., Lenkowicz J., Cellini F., Dinapoli N., Azario L., Teodoli S. (2019). Delta radiomics for rectal cancer response prediction with hybrid 0.35 T magnetic resonance-guided radiotherapy (MRgRT): A hypothesis-generating study for an innovative personalized medicine approach. Radiol. Med..

[29] Jeon S.H., Song C., Chie E.K., Kim B., Kim Y.H., Chang W., Lee Y.J., Chung J.H., Chung J.B., Lee K.W. (2019). Delta-radiomics signature predicts treatment outcomes after preoperative chemoradiotherapy and surgery in rectal cancer. Radiat. Oncol..

[30] Mazzei M.A., Nardone V., Di Giacomo L., Bagnacci G., Gentili F., Tini P., Marrelli D., Volterrani L. (2018). The role of delta radiomics in gastric cancer. Quant. Imaging Med. Surg..

[31] Nardone V., Reginelli A., Guida C., Belfiore M.P., Biondi M., Mormile M., Buonamici F.B., Di Giorgio E., Spadafora M., Tini P. (2020). Delta-radiomics increases multicentre reproducibility: A phantom study. Med. Oncol..

[32] Li H., Xu C., Xin B., Zheng C., Zhao Y., Hao K., Wang Q., Wahl R.L., Wang X., Zhou Y. (2019). (18)F-FDG PET/CT Radiomic Analysis with Machine Learning for Identifying Bone Marrow Involvement in the Patients with Suspected Relapsed Acute Leukemia. Theranostics.

[33] Reinert C.P., Federmann B., Hofmann J., Bösmüller H., Wirths S., Fritz J., Horger M. (2019). Computed tomography textural analysis for the differentiation of chronic lymphocytic leukemia and diffuse large B cell lymphoma of Richter syndrome. Eur. Radiol..

[34] Boldrini L., Bibault J.E., Masciocchi C., Shen Y., Bittner M.I. (2019). Deep Learning: A Review for the Radiation Oncologist. Front. Oncol..

[35] Alfieri R., Pintacuda G., Cagol M., Occhipinti T., Capraro I., Scarpa M., Zanchettin G., Cavallin F., Michelotto M., Giacomelli L. (2015). Oesophageal cancer: Assessment of tumour response to chemoradiotherapy with tridimensional CT. Radiol. Med..

[36] Giganti F., Salerno A., Ambrosi A., Chiari D., Orsenigo E., Esposito A., Albarello L., Mazza E., Staudacher C., Del Maschio A. (2016). Prognostic utility of diffusion-weighted MRI in oesophageal cancer: Is apparent diffusion coefficient a potential marker of tumour aggressiveness?. Radiol. Med..

[37] Sun N.N., Ge X.L., Liu X.S., Xu L.L. (2020). Histogram analysis of DCE-MRI for chemoradiotherapy response evaluation in locally advanced esophageal squamous cell carcinoma. Radiol. Med..

[38] Bi Y., Zhu X., Yu Z., Wu G., Han X. (2019). Interventional radiology protocol for treatment of esophagogastric anastomotic leakage. Radiol. Med..

[39] Bi Y., Zhu X., Yu Z., Jiao D., Yi M., Han X. (2020). Radioactive feeding tube in the palliation of esophageal malignant obstruction. Radiol. Med..

[40] Borghetti P., Bonu M.L., Giubbolini R., Levra N.G., Mazzola R., Perna M., Visani L., Meacci F., Taraborrelli M., Triggiani L. (2019). Concomitant radiotherapy and TKI in metastatic EGFR- or ALK-mutated non-small cell lung cancer: A multicentric analysis on behalf of AIRO lung cancer study group. Radiol. Med..

[41] Ganeshan B., Skogen K., Pressney I., Coutroubis D., Miles K. (2012). Tumour heterogeneity in oesophageal cancer assessed by CT texture analysis: Preliminary evidence of an association with tumour metabolism, stage, and survival. Clin. Radiol..

[42] Yip C., Landau D., Kozarski R., Ganeshan B., Thomas R., Michaelidou A., Goh V. (2014). Primary esophageal cancer: Heterogeneity as potential prognostic biomarker in patients treated with definitive chemotherapy and radiation therapy. Radiology.

[43] Yip C., Davnall F., Kozarski R., Landau D.B., Cook G.J., Ross P., Mason R., Goh V. (2015). Assessment of changes in tumor heterogeneity following neoadjuvant chemotherapy in primary esophageal cancer. Dis. Esophagus.

[44] Kwee R.M. (2010). Prediction of tumor response to neoadjuvant therapy in patients with esophageal cancer with use of 18F FDG PET: A systematic review. Radiology.

[45] Zhu W., Xing L., Yue J., Sun X., Sun X., Zhao H., Yu J. (2012). Prognostic significance of SUV on PET/CT in patients with localised oesophagogastric junction cancer receiving neoadjuvant chemotherapy/chemoradiation:a systematic review and meta-analysis. Br. J. Radiol..

[46] Zhang H., Tan S., Chen W., Kligerman S., Kim G., D−Souza W.D., Suntharalingam M., Lu W. (2014). Modeling pathologic response of esophageal cancer to chemoradiation therapy using spatial-temporal 18F-FDG PET features, clinical parameters, and demographics. Int. J. Radiat. Oncol. Biol. Phys..

[47] Dong X., Wu P., Sun X., Li W., Wan H., Yu J., Xing L. (2015). Intra-tumour 18F-FDG uptake heterogeneity decreases the reliability on target volume definition with positron emission tomography/computed tomography imaging. J. Med. Imaging Radiat. Oncol..

[48] Hatt M., Visvikis D., Pradier O., Cheze-le Rest C. (2011). Baseline (1)(8)F-FDG PET image-derived parameters for therapy response prediction in oesophageal cancer. Eur. J. Nucl. Med. Mol. Imaging.

[49] El Naqa I., Grigsby P., Apte A., Kidd E., Donnelly E., Khullar D., Chaudhari S., Yang D., Schmitt M., Laforest R. (2009). Exploring feature-based approaches in PET images for predicting cancer treatment outcomes. Pattern Recognit..

[50] Roedl J.B., Colen R.R., Holalkere N.S., Fischman A.J., Choi N.C., Blake M.A. (2008). Adenocarcinomas of the esophagus: Response to chemoradiotherapy is associated with decrease of metabolic tumor volume as measured on PET-CT. Comparison to histopathologic and clinical response evaluation. Radiother. Oncol..

[51] Blom R.L., Steenbakkers I.R., Lammering G., Vliegen R.F., Belgers E.J., de Jonge C., Schreurs W.M., Nap M., Sosef M.N. (2013). PET/CT-based metabolic tumour volume for response prediction of neoadjuvant chemoradiotherapy in oesophageal carcinoma. Eur. J. Nucl. Med. Mol. Imaging.

[52] van Rossum P.S., Fried D.V., Zhang L., Hofstetter W.L., van Vulpen M., Meijer G.J., Court L.E., Lin S.H. (2016). The Incremental Value of Subjective and Quantitative Assessment of 18F-FDG PET for the Prediction of Pathologic Complete Response to Preoperative Chemoradiotherapy in Esophageal Cancer. J. Nucl. Med..

[53] Simoni N., Rossi G., Benetti G., Zuffante M., Micera R., Pavarana M., Guariglia S., Zivelonghi E., Mengardo V., Weindelmayer J. (2020). (18)F-FDG PET/CT Metrics Are Correlated to the Pathological Response in Esophageal Cancer Patients Treated with Induction Chemotherapy Followed by Neoadjuvant Chemo-Radiotherapy. Front. Oncol..

[54] Yip S.S., Coroller T.P., Sanford N.N., Mamon H., Aerts H.J., Berbeco R.I. (2016). Relationship between the Temporal Changes in Positron-Emission-Tomography-Imaging-Based Textural Features and Pathologic Response and Survival in Esophageal Cancer Patients. Front. Oncol..

[55] Beukinga R.J., Hulshoff J.B., van Dijk L.V., Muijs C.T., Burgerhof J.G.M., Kats-Ugurlu G., Slart R., Slump C.H., Mul V.E.M., Plukker J.T.M. (2017). Predicting Response to Neoadjuvant Chemoradiotherapy in Esophageal Cancer with Textural Features Derived from Pretreatment (18)F-FDG PET/CT Imaging. J. Nucl. Med..

[56] Hatt M., Majdoub M., Vallieres M., Tixier F., Le Rest C.C., Groheux D., Hindie E., Martineau A., Pradier O., Hustinx R. (2015). 18F-FDG PET uptake characterization through texture analysis: Investigating the complementary nature of heterogeneity and functional tumor volume in a multi-cancer site patient cohort. J. Nucl. Med..

[57] Beukinga R.J., Wang D., Karrenbeld A., Dijksterhuis W.P.M., Faber H., Burgerhof J.G.M., Mul V.E.M., Slart R., Coppes R.P., Plukker J.T.M. (2021). Addition of HER2 and CD44 to (18)F-FDG PET-based clinico-radiomic models enhances prediction of neoadjuvant chemoradiotherapy response in esophageal cancer. Eur. Radiol..

[58] Nardone V., Pastina P., Giannicola R., Agostino R., Croci S., Tini P., Pirtoli L., Giordano A., Tagliaferri P., Correale P. (2018). How to Increase the Efficacy of Immunotherapy in NSCLC and HNSCC: Role of Radiation Therapy, Chemotherapy, and Other Strategies. Front. Immunol..

[59] Tini P., Nardone V., Pastina P., Pirtoli L., Correale P., Giordano A. (2018). The effects of radiotherapy on the survival of patients with unresectable non-small cell lung cancer. Expert Rev. Anticancer Ther..

[60] Parisi G., Mazzola R., Ciammella P., Timon G., Fozza A., Franceschini D., Navarria F., Bruni A., Perna M., Giaj-Levra N. (2019). Hypofractionated radiation therapy in the management of locally advanced NSCLC: A narrative review of the literature on behalf of the Italian Association of Radiation Oncology (AIRO)-Lung Working Group. Radiol. Med..

[61] Valeriani M., Marinelli L., Nicosia L., Reverberi C., De Sanctis V., Mollo D., Osti M.F. (2019). Locally advanced inoperable primary or recurrent non-small cell lung cancer treated with 4-week hypofractionated radiation therapy (3 Gy/fraction). Radiol. Med..

[62] Machado Medeiros T., Altmayer S., Watte G., Zanon M., Basso Dias A., Henz Concatto N., Hoefel Paes J., Mattiello R., de Souza Santos F., Mohammed T.L. (2020). 18F-FDG PET/CT and whole-body MRI diagnostic performance in M staging for non-small cell lung cancer: A systematic review and meta-analysis. Eur. Radiol..

[63] Pak K., Park S., Cheon G.J., Kang K.W., Kim I.J., Lee D.S., Kim E.E., Chung J.K. (2015). Update on nodal staging in non-small cell lung cancer with integrated positron emission tomography/computed tomography: A meta-analysis. Ann. Nucl. Med..

[64] Zhang Y., Ni J., Wei K., Tian J., Sun S. (2018). CT, MRI, and F-18 FDG PET for the detection of non-small-cell lung cancer (NSCLC): A protocol for a network meta-analysis of diagnostic test accuracy. Medicine.

[65] Arrigoni F., Bruno F., Zugaro L., Natella R., Cappabianca S., Russo U., Papapietro V.R., Splendiani A., Di Cesare E., Masciocchi C. (2018). Developments in the management of bone metastases with interventional radiology. Acta Biomed..

[66] Reginelli A., Silvestro G., Fontanella G., Sangiovanni A., Conte M., Nuzzo I., Calvanese M., Traettino M., Ferraioli P., Grassi R. (2016). Validation of DWI in assessment of radiotreated bone metastases in elderly patients. Int. J. Surg..

[67] Alessio N., Capasso S., Di Bernardo G., Cappabianca S., Casale F., Calarco A., Cipollaro M., Peluso G., Galderisi U. (2017). Mesenchymal stromal cells having inactivated RB1 survive following low irradiation and accumulate damaged DNA: Hints for side effects following radiotherapy. Cell Cycle.

[68] Nardone V., Tini P., Pastina P., Botta C., Reginelli A., Carbone S.F., Giannicola R., Calabrese G., Tebala C., Guida C. (2020). Radiomics predicts survival of patients with advanced non-small cell lung cancer undergoing PD-1 blockade using Nivolumab. Oncol. Lett..

[69] Franceschini D., Bruni A., Borghetti P., Giaj-Levra N., Ramella S., Buffoni L., Badellino S., Andolina M., Comin C., Vattemi E. (2020). Is multidisciplinary management possible in the treatment of lung cancer? A report from three Italian meetings. Radiol. Med..

[70] Phillips I., Ajaz M., Ezhil V., Prakash V., Alobaidli S., McQuaid S.J., South C., Scuffham J., Nisbet A., Evans P. (2018). Clinical applications of textural analysis in non-small cell lung cancer. Br. J. Radiol..

[71] Sollini M., Cozzi L., Antunovic L., Chiti A. (2017). PET Radiomics in NSCLC: State of the art and a proposal for harmonization of methodology. Sci. Rep..

[72] Rabbani M., Kanevsky J., Kafi K., Chandelier F., Giles F.J. (2018). Role of artificial intelligence in the care of patients with nonsmall cell lung cancer. Eur. J. Clin. Investig..

[73] Wong C.W., Chaudhry A. (2020). Radiogenomics of lung cancer. J. Thorac Dis.

[74] Shi L., He Y., Yuan Z., Benedict S., Valicenti R., Qiu J., Rong Y. (2018). Radiomics for Response and Outcome Assessment for Non-Small Cell Lung Cancer. Technol. Cancer Res. Treat..

[75] Ninatti G., Kirienko M., Neri E., Sollini M., Chiti A. (2020). Imaging-Based Prediction of Molecular Therapy Targets in NSCLC by Radiogenomics and AI Approaches: A Systematic Review. Diagnostics.

[76] Reginelli A., Di Grezia G., Izzo A., D’Andrea A., Gatta G., Cappabianca S., Squillaci E., Grassi R. (2014). Imaging of adrenal incidentaloma: Our experience. Int. J. Surg..

[77] Sun J., Hu D., Shen Y., Yang H., Chen C., Yin J., Peng Y. (2019). Improving image quality with model-based iterative reconstruction algorithm for chest CT in children with reduced contrast concentration. Radiol. Med..

[78] Chang C., Sun X., Zhao W., Wang R., Qian X., Lei B., Wang L., Liu L., Ruan M., Xie W. (2020). Minor components of micropapillary and solid subtypes in lung invasive adenocarcinoma (≤3 cm): PET/CT findings and correlations with lymph node metastasis. Radiol. Med..

[79] Zhang G., Yang Z., Gong L., Jiang S. (2020). Classification of lung nodules based on CT images using squeeze-and-excitation network and aggregated residual transformations. Radiol. Med..

[80] Albain K.S., Swann R.S., Rusch V.W., Turrisi A.T., Shepherd F.A., Smith C., Chen Y., Livingston R.B., Feins R.H., Gandara D.R. (2009). Radiotherapy plus chemotherapy with or without surgical resection for stage III non-small-cell lung cancer: A phase III randomised controlled trial. Lancet.

[81] van Meerbeeck J.P., Kramer G.W., Van Schil P.E., Legrand C., Smit E.F., Schramel F., Tjan-Heijnen V.C., Biesma B., Debruyne C., van Zandwijk N. (2007). Randomized controlled trial of resection versus radiotherapy after induction chemotherapy in stage IIIA-N2 non-small-cell lung cancer. J. Natl. Cancer Inst..

[82] Coroller T.P., Agrawal V., Narayan V., Hou Y., Grossmann P., Lee S.W., Mak R.H., Aerts H.J. (2016). Radiomic phenotype features predict pathological response in non-small cell lung cancer. Radiother. Oncol..

[83] Coroller T.P., Agrawal V., Huynh E., Narayan V., Lee S.W., Mak R.H., Aerts H. (2017). Radiomic-Based Pathological Response Prediction from Primary Tumors and Lymph Nodes in NSCLC. J. Thorac. Oncol..

[84] Kozak M.M., Murphy J.D., Schipper M.L., Donington J.S., Zhou L., Whyte R.I., Shrager J.B., Hoang C.D., Bazan J., Maxim P.G. (2011). Tumor volume as a potential imaging-based risk-stratification factor in trimodality therapy for locally advanced non-small cell lung cancer. J. Thorac. Oncol..

[85] Chong Y., Kim J.H., Lee H.Y., Ahn Y.C., Lee K.S., Ahn M.J., Kim J., Shim Y.M., Han J., Choi Y.L. (2014). Quantitative CT variables enabling response prediction in neoadjuvant therapy with EGFR-TKIs: Are they different from those in neoadjuvant concurrent chemoradiotherapy?. PLoS ONE.

[86] Khorrami M., Jain P., Bera K., Alilou M., Thawani R., Patil P., Ahmad U., Murthy S., Stephans K., Fu P. (2019). Predicting pathologic response to neoadjuvant chemoradiation in resectable stage III non-small cell lung cancer patients using computed tomography radiomic features. Lung Cancer.

[87] Sbaraglia M., Dei Tos A.P. (2019). The pathology of soft tissue sarcomas. Radiol. Med..

[88] Badalamenti G., Messina C., De Luca I., Musso E., Casarin A., Incorvaia L. (2019). Soft tissue sarcomas in the precision medicine era: New advances in clinical practice and future perspectives. Radiol. Med..

[89] Greto D., Loi M., Terziani F., Visani L., Garlatti P., Lo Russo M., Teriaca A., Muntoni C., Delli Paoli C., Topulli J. (2019). A matched cohort study of radio-chemotherapy versus radiotherapy alone in soft tissue sarcoma patients. Radiol. Med..

[90] Greto D., Saieva C., Loi M., Terziani F., Visani L., Garlatti P., Lo Russo M., Muntoni C., Becherini C., Topulli J. (2019). Influence of age and subtype in outcome of operable liposarcoma. Radiol. Med..

[91] Strauss D.C., Qureshi Y.A., Hayes A.J., Thway K., Fisher C., Thomas J.M. (2010). The role of core needle biopsy in the diagnosis of suspected soft tissue tumours. J. Surg. Oncol..

[92] Dangoor A., Seddon B., Gerrand C., Grimer R., Whelan J., Judson I. (2016). UK guidelines for the management of soft tissue sarcomas. Clin. Sarcoma Res..

[93] Greto D., Livi L., Saieva C., Bonomo P., Meattini I., Loi M., Di Brina L., Beltrami G., Campanacci D., Scoccianti G. (2014). Neoadjuvant treatment of soft tissue sarcoma. Radiol. Med..

[94] Mangoni M., Sottili M., Salvatore G., Campanacci D., Scoccianti G., Beltrami G., Paoli C.D., Dominici L., Maragna V., Olmetto E. (2019). Soft tissue sarcomas: New opportunity of treatment with PARP inhibitors?. Radiol. Med..

[95] Pasquali S., Colombo C., Pizzamiglio S., Verderio P., Callegaro D., Stacchiotti S., Martin Broto J., Lopez-Pousa A., Ferrari S., Poveda A. (2018). High-risk soft tissue sarcomas treated with perioperative chemotherapy: Improving prognostic classification in a randomised clinical trial. Eur. J. Cancer.

[96] Chibon F., Lagarde P., Salas S., Perot G., Brouste V., Tirode F., Lucchesi C., de Reynies A., Kauffmann A., Bui B. (2010). Validated prediction of clinical outcome in sarcomas and multiple types of cancer on the basis of a gene expression signature related to genome complexity. Nat. Med..

[97] Robba T., Chianca V., Albano D., Clementi V., Piana R., Linari A., Comandone A., Regis G., Stratta M., Faletti C. (2017). Diffusion-weighted imaging for the cellularity assessment and matrix characterization of soft tissue tumour. Radiol. Med..

[98] Fields B.K.K., Demirjian N.L., Hwang D.H., Varghese B.A., Cen S.Y., Lei X., Desai B., Duddalwar V., Matcuk G.R. (2021). Whole-tumor 3D volumetric MRI-based radiomics approach for distinguishing between benign and malignant soft tissue tumors. Eur. Radiol..

[99] Leporq B., Bouhamama A., Pilleul F., Lame F., Bihane C., Sdika M., Blay J.Y., Beuf O. (2020). MRI-based radiomics to predict lipomatous soft tissue tumors malignancy: A pilot study. Cancer Imaging: Off. Publ. Int. Cancer Imaging Soc..

[100] Vos M., Starmans M.P.A., Timbergen M.J.M., van der Voort S.R., Padmos G.A., Kessels W., Niessen W.J., van Leenders G., Grunhagen D.J., Sleijfer S. (2019). Radiomics approach to distinguish between well differentiated liposarcomas and lipomas on MRI. Br. J. Surg..

[101] Malinauskaite I., Hofmeister J., Burgermeister S., Neroladaki A., Hamard M., Montet X., Boudabbous S. (2020). Radiomics and Machine Learning Differentiate Soft-Tissue Lipoma and Liposarcoma Better than Musculoskeletal Radiologists. Sarcoma.

[102] Martin-Carreras T., Li H., Cooper K., Fan Y., Sebro R. (2019). Radiomic features from MRI distinguish myxomas from myxofibrosarcomas. BMC Med. Imaging.

[103] Xie H., Hu J., Zhang X., Ma S., Liu Y., Wang X. (2019). Preliminary utilization of radiomics in differentiating uterine sarcoma from atypical leiomyoma: Comparison on diagnostic efficacy of MRI features and radiomic features. Eur. J. Radiol..

[104] Xie H., Zhang X., Ma S., Liu Y., Wang X. (2019). Preoperative Differentiation of Uterine Sarcoma from Leiomyoma: Comparison of Three Models Based on Different Segmentation Volumes Using Radiomics. Mol. Imaging Biol..

[105] Wang T., Gong J., Li Q., Chu C., Shen W., Peng W., Gu Y., Li W. (2021). A combined radiomics and clinical variables model for prediction of malignancy in T2 hyperintense uterine mesenchymal tumors on MRI. Eur. Radiol..

[106] Yan R., Hao D., Li J., Liu J., Hou F., Chen H., Duan L., Huang C., Wang H., Yu T. (2021). Magnetic Resonance Imaging-Based Radiomics Nomogram for Prediction of the Histopathological Grade of Soft Tissue Sarcomas: A Two-Center Study. J. Magn. Reason. Imaging.

[107] Xu W., Hao D., Hou F., Zhang D., Wang H. (2020). Soft Tissue Sarcoma: Preoperative MRI-Based Radiomics and Machine Learning May Be Accurate Predictors of Histopathologic Grade. AJR Am. J. Roentgenol.

[108] Peeken J.C., Spraker M.B., Knebel C., Dapper H., Pfeiffer D., Devecka M., Thamer A., Shouman M.A., Ott A., von Eisenhart-Rothe R. (2019). Tumor grading of soft tissue sarcomas using MRI-based radiomics. EBioMedicine.

[109] Wang H., Chen H., Duan S., Hao D., Liu J. (2020). Radiomics and Machine Learning with Multiparametric Preoperative MRI May Accurately Predict the Histopathological Grades of Soft Tissue Sarcomas. J. Magn. Reason. Imaging.

[110] Peeken J.C., Bernhofer M., Spraker M.B., Pfeiffer D., Devecka M., Thamer A., Shouman M.A., Ott A., Nusslin F., Mayr N.A. (2019). CT-based radiomic features predict tumor grading and have prognostic value in patients with soft tissue sarcomas treated with neoadjuvant radiation therapy. Radiother. Oncol..

[111] Zhang Y., Zhu Y., Shi X., Tao J., Cui J., Dai Y., Zheng M., Wang S. (2019). Soft Tissue Sarcomas: Preoperative Predictive Histopathological Grading Based on Radiomics of MRI. Acad. Radiol..

[112] Corino V.D.A., Montin E., Messina A., Casali P.G., Gronchi A., Marchiano A., Mainardi L.T. (2018). Radiomic analysis of soft tissues sarcomas can distinguish intermediate from high-grade lesions. J. Magn. Reason. Imaging.

[113] Tian L., Zhang D., Bao S., Nie P., Hao D., Liu Y., Zhang J., Wang H. (2021). Radiomics-based machine-learning method for prediction of distant metastasis from soft-tissue sarcomas. Clin. Radiol..

[114] Crombe A., Le Loarer F., Sitbon M., Italiano A., Stoeckle E., Buy X., Kind M. (2020). Can radiomics improve the prediction of metastatic relapse of myxoid/round cell liposarcomas?. Eur. Radiol..

[115] Peng Y., Bi L., Guo Y., Feng D., Fulham M., Kim J. Deep multi-modality collaborative learning for distant metastases predication in PET-CT soft-tissue sarcoma studies. Proceedings of the 41st Annual International Conference of the IEEE Engineering in Medicine and Biology Society.

[116] Vallieres M., Freeman C.R., Skamene S.R., El Naqa I. (2015). A radiomics model from joint FDG-PET and MRI texture features for the prediction of lung metastases in soft-tissue sarcomas of the extremities. Phys. Med. Biol..

[117] Peeken J.C., Neumann J., Asadpour R., Leonhardt Y., Moreira J.R., Hippe D.S., Klymenko O., Foreman S.C., von Schacky C.E., Spraker M.B. (2021). Prognostic Assessment in High-Grade Soft-Tissue Sarcoma Patients: A Comparison of Semantic Image Analysis and Radiomics. Cancers.

[118] Spraker M.B., Wootton L.S., Hippe D.S., Ball K.C., Peeken J.C., Macomber M.W., Chapman T.R., Hoff M.N., Kim E.Y., Pollack S.M. (2019). MRI Radiomic Features Are Independently Associated With Overall Survival in Soft Tissue Sarcoma. Adv. Radiat. Oncol..

[119] Esser M., Kloth C., Thaiss W.M., Reinert C.P., Fritz J., Kopp H.G., Horger M. (2018). CT-response patterns and the role of CT-textural features in inoperable abdominal/retroperitoneal soft tissue sarcomas treated with trabectedin. Eur. J. Radiol..

[120] Tagliafico A.S., Bignotti B., Rossi F., Valdora F., Martinoli C. (2019). Local recurrence of soft tissue sarcoma: A radiomic analysis. Radiol. Oncol..

[121] Crombe A., Perier C., Kind M., De Senneville B.D., Le Loarer F., Italiano A., Buy X., Saut O. (2019). T2 -based MRI Delta-radiomics improve response prediction in soft-tissue sarcomas treated by neoadjuvant chemotherapy. J. Magn. Reason. Imaging.

[122] Tian F., Hayano K., Kambadakone A.R., Sahani D.V. (2015). Response assessment to neoadjuvant therapy in soft tissue sarcomas: Using CT texture analysis in comparison to tumor size, density, and perfusion. Abdom. Imaging.

[123] Esser M., Kloth C., Thaiss W.M., Reinert C.P., Kraus M.S., Gast G.C., Horger M. (2019). CT-morphologic and CT-textural patterns of response in inoperable soft tissue sarcomas treated with pazopanib-a preliminary retrospective cohort study. Br. J. Radiol..

[124] Crombe A., Sitbon M., Stoeckle E., Italiano A., Buy X., Le Loarer F., Kind M. (2020). Magnetic resonance imaging assessment of chemotherapy-related adipocytic maturation in myxoid/round cell liposarcomas: Specificity and prognostic value. Br. J. Radiol..

[125] Gao Y., Kalbasi A., Hsu W., Ruan D., Fu J., Shao J., Cao M., Wang C., Eilber F.C., Bernthal N. (2020). Treatment effect prediction for sarcoma patients treated with preoperative radiotherapy using radiomics features from longitudinal diffusion-weighted MRIs. Phys. Med. Biol..

[126] Crombe A., Fadli D., Italiano A., Saut O., Buy X., Kind M. (2020). Systematic review of sarcomas radiomics studies: Bridging the gap between concepts and clinical applications?. Eur J. Radiol..

[127] Schmoll H.J., Van Cutsem E., Stein A., Valentini V., Glimelius B., Haustermans K., Nordlinger B., van de Velde C.J., Balmana J., Regula J. (2012). ESMO Consensus Guidelines for management of patients with colon and rectal cancer. a personalized approach to clinical decision making. Ann. Oncol..

[128] Liu X., Zheng S., Peng Y., Zhuang J., Yang Y., Xu Y., Guan G. (2021). Construction of the Prediction Model for Locally Advanced Rectal Cancer Following Neoadjuvant Chemoradiotherapy Based on Pretreatment Tumor-Infiltrating Macrophage-Associated Biomarkers. OncoTargets Ther..

[129] Hamid H.K.S., Davis G.N., Trejo-Avila M., Igwe P.O., Garcia-Marin A. (2021). Prognostic and predictive value of neutrophil-to-lymphocyte ratio after curative rectal cancer resection: A systematic review and meta-analysis. Surg. Oncol..

[130] Yi Y., Shen L., Shi W., Xia F., Zhang H., Wang Y., Zhang J., Wang Y., Sun X., Zhang Z. (2021). Gut Microbiome Components Predict Response to Neoadjuvant Chemoradiotherapy in Patients with Locally Advanced Rectal Cancer: A Prospective, Longitudinal Study. Clin. Cancer Res..

[131] Spatola C., Privitera G., Milazzotto R., Tocco A., Acquaviva G., Marletta F., Marino L., Di Grazia A., Salvo R., Cartia G. (2019). Trends in combined radio-chemotherapy for locally advanced rectal cancer: A survey among radiation oncology centers of Sicily region on behalf of AIRO. Radiol. Med..

[132] Ciolina M., Caruso D., De Santis D., Zerunian M., Rengo M., Alfieri N., Musio D., De Felice F., Ciardi A., Tombolini V. (2019). Dynamic contrast-enhanced magnetic resonance imaging in locally advanced rectal cancer: Role of perfusion parameters in the assessment of response to treatment. Radiol. Med..

[133] Bertocchi E., Barugola G., Nicosia L., Mazzola R., Ricchetti F., Dell−Abate P., Alongi F., Ruffo G. (2020). A comparative analysis between radiation dose intensification and conventional fractionation in neoadjuvant locally advanced rectal cancer: A monocentric prospective observational study. Radiol. Med..

[134] Kim Y.H., Kim D.Y., Kim T.H., Jung K.H., Chang H.J., Jeong S.Y., Sohn D.K., Choi H.S., Ahn J.B., Kim D.H. (2005). Usefulness of magnetic resonance volumetric evaluation in predicting response to preoperative concurrent chemoradiotherapy in patients with resectable rectal cancer. Int. J. Radiat. Oncol. Biol. Phys..

[135] Wieder H.A., Rosenberg R., Lordick F., Geinitz H., Beer A., Becker K., Woertler K., Dobritz M., Siewert J.R., Rummeny E.J. (2007). Rectal cancer: MR imaging before neoadjuvant chemotherapy and radiation therapy for prediction of tumor-free circumferential resection margins and long-term survival. Radiology.

[136] Barbaro B., Fiorucci C., Tebala C., Valentini V., Gambacorta M.A., Vecchio F.M., Rizzo G., Coco C., Crucitti A., Ratto C. (2009). Locally advanced rectal cancer: MR imaging in prediction of response after preoperative chemotherapy and radiation therapy. Radiology.

[137] Cascini G.L., Avallone A., Delrio P., Guida C., Tatangelo F., Marone P., Aloj L., De Martinis F., Comella P., Parisi V. (2006). 18F-FDG PET is an early predictor of pathologic tumor response to preoperative radiochemotherapy in locally advanced rectal cancer. J. Nucl. Med..

[138] Rosenberg R., Herrmann K., Gertler R., Kunzli B., Essler M., Lordick F., Becker K., Schuster T., Geinitz H., Maak M. (2009). The predictive value of metabolic response to preoperative radiochemotherapy in locally advanced rectal cancer measured by PET/CT. Int. J. Color. Dis.

[139] Coppola F., Giannini V., Gabelloni M., Panic J., Defeudis A., Lo Monaco S., Cattabriga A., Cocozza M.A., Pastore L.V., Polici M. (2021). Radiomics and Magnetic Resonance Imaging of Rectal Cancer: From Engineering to Clinical Practice. Diagnostics.

[140] Fischer J., Eglinton T.W., Richards S.J., Frizelle F.A. (2021). Predicting pathological response to chemoradiotherapy for rectal cancer: A systematic review. Expert Rev. Anticancer Ther..

[141] Staal F.C.R., van der Reijd D.J., Taghavi M., Lambregts D.M.J., Beets-Tan R.G.H., Maas M. (2021). Radiomics for the Prediction of Treatment Outcome and Survival in Patients with Colorectal Cancer: A Systematic Review. Clin. Color. Cancer.

[142] Whiting P.F., Rutjes A.W., Westwood M.E., Mallett S., Deeks J.J., Reitsma J.B., Leeflang M.M., Sterne J.A., Bossuyt P.M., Group Q. (2011). QUADAS-2: A revised tool for the quality assessment of diagnostic accuracy studies. Ann. Intern. Med..

[143] Lambin P., Leijenaar R.T.H., Deist T.M., Peerlings J., de Jong E.E.C., van Timmeren J., Sanduleanu S., Larue R., Even A.J.G., Jochems A. (2017). Radiomics: The bridge between medical imaging and personalized medicine. Nat. Rev. Clin. Oncol..

[144] Al-Sukhni E., Milot L., Fruitman M., Beyene J., Victor J.C., Schmocker S., Brown G., McLeod R., Kennedy E. (2012). Diagnostic accuracy of MRI for assessment of T category, lymph node metastases, and circumferential resection margin involvement in patients with rectal cancer: A systematic review and meta-analysis. Ann. Surg. Oncol..

[145] Petresc B., Lebovici A., Caraiani C., Feier D.S., Graur F., Buruian M.M. (2020). Pre-Treatment T2-WI Based Radiomics Features for Prediction of Locally Advanced Rectal Cancer Non-Response to Neoadjuvant Chemoradiotherapy: A Preliminary Study. Cancers.

[146] Antunes J.T., Ofshteyn A., Bera K., Wang E.Y., Brady J.T., Willis J.E., Friedman K.A., Marderstein E.L., Kalady M.F., Stein S.L. (2020). Radiomic Features of Primary Rectal Cancers on Baseline T(2)—Weighted MRI Are Associated With Pathologic Complete Response to Neoadjuvant Chemoradiation: A Multisite Study. J. Magn. Reson. Imaging.

[147] Ferrari R., Mancini-Terracciano C., Voena C., Rengo M., Zerunian M., Ciardiello A., Grasso S., Mare V., Paramatti R., Russomando A. (2019). MR-based artificial intelligence model to assess response to therapy in locally advanced rectal cancer. Eur. J. Radiol..

[148] Fu J., Zhong X., Li N., Van Dams R., Lewis J., Sung K., Raldow A.C., Jin J., Qi X.S. (2020). Deep learning-based radiomic features for improving neoadjuvant chemoradiation response prediction in locally advanced rectal cancer. Phys. Med. Biol..

[149] Jalil O., Afaq A., Ganeshan B., Patel U.B., Boone D., Endozo R., Groves A., Sizer B., Arulampalam T. (2017). Magnetic resonance based texture parameters as potential imaging biomarkers for predicting long-term survival in locally advanced rectal cancer treated by chemoradiotherapy. Color. Dis..

[150] Cusumano D., Boldrini L., Yadav P., Yu G., Musurunu B., Chiloiro G., Piras A., Lenkowicz J., Placidi L., Romano A. (2021). Delta radiomics for rectal cancer response prediction using low field magnetic resonance guided radiotherapy: An external validation. Phys. Med..

[151] Liang H.Y., Huang Y.Q., Yang Z.X., Ying D., Zeng M.S., Rao S.X. (2016). Potential of MR histogram analyses for prediction of response to chemotherapy in patients with colorectal hepatic metastases. Eur. Radiol..

[152] Cui Y., Yang X., Shi Z., Yang Z., Du X., Zhao Z., Cheng X. (2019). Radiomics analysis of multiparametric MRI for prediction of pathological complete response to neoadjuvant chemoradiotherapy in locally advanced rectal cancer. Eur. Radiol..

[153] Cusumano D., Dinapoli N., Boldrini L., Chiloiro G., Gatta R., Masciocchi C., Lenkowicz J., Casa C., Damiani A., Azario L. (2018). Fractal-based radiomic approach to predict complete pathological response after chemo-radiotherapy in rectal cancer. Radiol. Med..

[154] Dinapoli N., Barbaro B., Gatta R., Chiloiro G., Casa C., Masciocchi C., Damiani A., Boldrini L., Gambacorta M.A., Dezio M. (2018). Magnetic Resonance, Vendor-independent, Intensity Histogram Analysis Predicting Pathologic Complete Response After Radiochemotherapy of Rectal Cancer. Int. J. Radiat. Oncol. Biol. Phys..

[155] Liu Y., Zhang F.J., Zhao X.X., Yang Y., Liang C.Y., Feng L.L., Wan X.B., Ding Y., Zhang Y.W. (2021). Development of a Joint Prediction Model Based on Both the Radiomics and Clinical Factors for Predicting the Tumor Response to Neoadjuvant Chemoradiotherapy in Patients with Locally Advanced Rectal Cancer. Cancer Manag. Res..

[156] Caruso D., Zerunian M., Ciolina M., de Santis D., Rengo M., Soomro M.H., Giunta G., Conforto S., Schmid M., Neri E. (2018). Haralick−s texture features for the prediction of response to therapy in colorectal cancer: A preliminary study. Radiol. Med..

[157] Li Z.Y., Wang X.D., Li M., Liu X.J., Ye Z., Song B., Yuan F., Yuan Y., Xia C.C., Zhang X. (2020). Multi-modal radiomics model to predict treatment response to neoadjuvant chemotherapy for locally advanced rectal cancer. World J. Gastroenterol..

[158] Liang M., Cai Z., Zhang H., Huang C., Meng Y., Zhao L., Li D., Ma X., Zhao X. (2019). Machine Learning-based Analysis of Rectal Cancer MRI Radiomics for Prediction of Metachronous Liver Metastasis. Acad. Radiol..

[159] Liu M., Lv H., Liu L.H., Yang Z.H., Jin E.H., Wang Z.C. (2017). Locally advanced rectal cancer: Predicting non-responders to neoadjuvant chemoradiotherapy using apparent diffusion coefficient textures. Int. J. Color. Dis..

[160] Meng Y., Zhang C., Zou S., Zhao X., Xu K., Zhang H., Zhou C. (2018). MRI texture analysis in predicting treatment response to neoadjuvant chemoradiotherapy in rectal cancer. Oncotarget.

[161] Meng Y., Zhang Y., Dong D., Li C., Liang X., Zhang C., Wan L., Zhao X., Xu K., Zhou C. (2018). Novel radiomic signature as a prognostic biomarker for locally advanced rectal cancer. J. Magn. Reason. Imaging.

[162] De Cecco C.N., Ganeshan B., Ciolina M., Rengo M., Meinel F.G., Musio D., De Felice F., Raffetto N., Tombolini V., Laghi A. (2015). Texture analysis as imaging biomarker of tumoral response to neoadjuvant chemoradiotherapy in rectal cancer patients studied with 3-T magnetic resonance. Investig. Radiol..

[163] De Cecco C.N., Ciolina M., Caruso D., Rengo M., Ganeshan B., Meinel F.G., Musio D., De Felice F., Tombolini V., Laghi A. (2016). Performance of diffusion-weighted imaging, perfusion imaging, and texture analysis in predicting tumoral response to neoadjuvant chemoradiotherapy in rectal cancer patients studied with 3T MR: Initial experience. Abdom. Radiol..

[164] Nardone V., Reginelli A., Scala F. (2019). Magnetic-Resonance-Imaging Texture Analysis Predicts Early Progression in Rectal Cancer Patients Undergoing Neoadjuvant Chemoradiation. Gastroenterol. Res. Pract..

[165] Nie K., Shi L., Chen Q., Hu X., Jabbour S.K., Yue N., Niu T., Sun X. (2016). Rectal Cancer: Assessment of Neoadjuvant Chemoradiation Outcome based on Radiomics of Multiparametric MRI. Clin. Cancer Res..

[166] Nougaret S., Vargas H.A., Lakhman Y., Sudre R., Do R.K., Bibeau F., Azria D., Assenat E., Molinari N., Pierredon M.A. (2016). Intravoxel Incoherent Motion-derived Histogram Metrics for Assessment of Response after Combined Chemotherapy and Radiation Therapy in Rectal Cancer: Initial Experience and Comparison between Single-Section and Volumetric Analyses. Radiology.

[167] Palmisano A., Esposito A., Rancoita P.M.V., Di Chiara A., Passoni P., Slim N., Campolongo M., Albarello L., Fiorino C., Rosati R. (2018). Could perfusion heterogeneity at dynamic contrast-enhanced MRI be used to predict rectal cancer sensitivity to chemoradiotherapy?. Clin. Radiol..

[168] Park H., Kim K.A., Jung J.H., Rhie J., Choi S.Y. (2020). MRI features and texture analysis for the early prediction of therapeutic response to neoadjuvant chemoradiotherapy and tumor recurrence of locally advanced rectal cancer. Eur. Radiol..

[169] Petkovska I., Tixier F., Ortiz E.J., Golia Pernicka J.S., Paroder V., Bates D.D., Horvat N., Fuqua J., Schilsky J., Gollub M.J. (2020). Clinical utility of radiomics at baseline rectal MRI to predict complete response of rectal cancer after chemoradiation therapy. Abdom. Radiol..

[170] Shaish H., Aukerman A., Vanguri R., Spinelli A., Armenta P., Jambawalikar S., Makkar J., Bentley-Hibbert S., Del Portillo A., Kiran R. (2020). Radiomics of MRI for pretreatment prediction of pathologic complete response, tumor regression grade, and neoadjuvant rectal score in patients with locally advanced rectal cancer undergoing neoadjuvant chemoradiation: An international multicenter study. Eur. Radiol..

[171] Crimì F., Capelli G., Spolverato G., Bao Q.R., Florio A., Milite Rossi S., Cecchin D., Albertoni L., Campi C., Pucciarelli S. (2020). MRI T2-weighted sequences-based texture analysis (TA) as a predictor of response to neoadjuvant chemo-radiotherapy (nCRT) in patients with locally advanced rectal cancer (LARC). Radiol. Med..

[172] Fornell-Perez R., Vivas-Escalona V., Aranda-Sanchez J., Gonzalez-Dominguez M.C., Rubio-Garcia J., Aleman-Flores P., Lozano-Rodriguez A., Porcel-de-Peralta G., Loro-Ferrer J.F. (2020). Primary and post-chemoradiotherapy MRI detection of extramural venous invasion in rectal cancer: The role of diffusion-weighted imaging. Radiol. Med..

[173] Boldrini L., Intven M., Bassetti M., Valentini V., Gani C. (2021). MR-Guided Radiotherapy for Rectal Cancer: Current Perspective on Organ Preservation. Front. Oncol..

[174] Chiloiro G., Boldrini L., Meldolesi E., Re A., Cellini F., Cusumano D., Corvari B., Mantini G., Balducci M., Valentini V. (2019). MR-guided radiotherapy in rectal cancer: First clinical experience of an innovative technology. Clin. Transl. Radiat. Oncol..

[175] Gani C., Boldrini L., Valentini V. (2019). Online MR guided radiotherapy for rectal cancer. New opportunities. Clin. Transl. Radiat. Oncol..

[176] Bundschuh R.A., Dinges J., Neumann L., Seyfried M., Zsoter N., Papp L., Rosenberg R., Becker K., Astner S.T., Henninger M. (2014). Textural Parameters of Tumor Heterogeneity in (1)(8)F-FDG PET/CT for Therapy Response Assessment and Prognosis in Patients with Locally Advanced Rectal Cancer. J. Nucl. Med..

[177] Bang J.I., Ha S., Kang S.B., Lee K.W., Lee H.S., Kim J.S., Oh H.K., Lee H.Y., Kim S.E. (2016). Prediction of neoadjuvant radiation chemotherapy response and survival using pretreatment [(18)F]FDG PET/CT scans in locally advanced rectal cancer. Eur. J. Nucl. Med. Mol. Imaging.

[178] Lovinfosse P., Polus M., Van Daele D., Martinive P., Daenen F., Hatt M., Visvikis D., Koopmansch B., Lambert F., Coimbra C. (2018). FDG PET/CT radiomics for predicting the outcome of locally advanced rectal cancer. Eur. J. Nucl. Med. Mol. Imaging.

[179] Yuan Z., Frazer M., Rishi A., Latifi K., Tomaszewski M.R., Moros E.G., Feygelman V., Felder S., Sanchez J., Dessureault S. (2021). Pretreatment CT and PET radiomics predicting rectal cancer patients in response to neoadjuvant chemoradiotherapy. Rep. Pract. Oncol. Radiother..

[180] Giannini V., Mazzetti S., Bertotto I., Chiarenza C., Cauda S., Delmastro E., Bracco C., Di Dia A., Leone F., Medico E. (2019). Predicting locally advanced rectal cancer response to neoadjuvant therapy with (18)F-FDG PET and MRI radiomics features. Eur. J. Nucl. Med. Mol. Imaging.

[181] Ree A.H., Redalen K.R. (2015). Personalized radiotherapy: Concepts, biomarkers and trial design. Br. J. Radiol..

[182] Cesario A., D−Oria M., Calvani R., Picca A., Pietragalla A., Lorusso D., Daniele G., Lohmeyer F.M., Boldrini L., Valentini V. (2021). The Role of Artificial Intelligence in Managing Multimorbidity and Cancer. J. Pers. Med..

[183] Bogowicz M., Riesterer O., Bundschuh R.A., Veit-Haibach P., Hüllner M., Studer G., Stieb S., Glatz S., Pruschy M., Guckenberger M. (2016). Stability of radiomic features in CT perfusion maps. Phys. Med. Biol..

[184] Larue R.T., Defraene G., De Ruysscher D., Lambin P., van Elmpt W. (2017). Quantitative radiomics studies for tissue characterization: A review of technology and methodological procedures. Br. J. Radiol..

[185] Espinasse M., Pitre-Champagnat S., Charmettant B., Bidault F., Volk A., Balleyguier C., Lassau N., Caramella C. (2020). CT Texture Analysis Challenges: Influence of Acquisition and Reconstruction Parameters: A Comprehensive Review. Diagnostics.

[186] Orlhac F., Boughdad S., Philippe C., Stalla-Bourdillon H., Nioche C., Champion L., Soussan M., Frouin F., Frouin V., Buvat I. (2018). A Postreconstruction Harmonization Method for Multicenter Radiomic Studies in PET. J. Nucl. Med..

[187] Choe J., Lee S.M. (2019). Deep Learning-based Image Conversion of CT Reconstruction Kernels Improves Radiomics Reproducibility for Pulmonary Nodules or Masses. Radiology.

[188] Parmar C., Grossmann P., Bussink J., Lambin P., Aerts H. (2015). Machine Learning methods for Quantitative Radiomic Biomarkers. Sci. Rep..

[189] Avanzo M., Wei L., Stancanello J., Vallières M., Rao A., Morin O., Mattonen S.A., El Naqa I. (2020). Machine and deep learning methods for radiomics. Med. Phys..

[190] Desideri I., Loi M., Francolini G., Becherini C., Livi L., Bonomo P. (2020). Application of Radiomics for the Prediction of Radiation-Induced Toxicity in the IMRT Era: Current State-of-the-Art. Front. Oncol..

[191] Francolini G., Desideri I., Stocchi G., Salvestrini V., Ciccone L.P., Garlatti P., Loi M., Livi L. (2020). Artificial Intelligence in radiotherapy: State of the art and future directions. Med. Oncol..

[192] Verma V., Simone C.B., Krishnan S., Lin S.H., Yang J., Hahn S.M. (2017). The Rise of Radiomics and Implications for Oncologic Management. J. Natl. Cancer Inst..

[193] Limkin E.J., Sun R., Dercle L., Zacharaki E.I., Robert C., Reuzé S., Schernberg A., Paragios N., Deutsch E., Ferté C. (2017). Promises and challenges for the implementation of computational medical imaging (radiomics) in oncology. Ann. Oncol..

[194] Teo Y.Y.A., Danilevsky A., Shomron N. (2021). Overcoming Interpretability in Deep Learning Cancer Classification. Methods Mol. Biol..

